# Chemical Synthesis, Efficacy, and Safety of Antimalarial Hybrid Drug Comprising of Sarcosine and Aniline Pharmacophores as Scaffolds

**DOI:** 10.1155/2020/1643015

**Published:** 2020-04-09

**Authors:** Jean Baptiste Niyibizi, Peter G. Kirira, Francis T. Kimani, Fiona Oyatsi, Joseph K. Ng'ang'a

**Affiliations:** ^1^Pan African University Institute of Sciences, Technology and Innovation, Department of Molecular Biology and Biotechnology, Nairobi, Kenya; ^2^University of Global Health Equity, MBBS/Basic Medical Sciences Division, Butaro/Kigali, Rwanda; ^3^Mount Kenya University, Department of Physical Chemistry, Nairobi, Kenya; ^4^Kenya Medical Research Institute, Nairobi, Kenya; ^5^Jomo Kenyatta University of Agriculture and Technology, School of Biomedical Sciences, Biochemistry Department, Nairobi, Kenya

## Abstract

Malaria is a disease caused by protozoans transmitted to humans by infected female *Anopheles* mosquitoes. According to the WHO report of 2015, there were 214 million cases of malaria with 438,000 deaths worldwide. Ninety percent of world's malaria cases occur in Africa, where the disease is recognized as a serious impediment to economic and social development. Despite advancement in malaria research, the disease continues to be a global problem, especially in developing countries. Currently, there is no effective vaccine for malaria control. In addition, although there are effective drugs for treatment of malaria, this could be lost to the drug resistance in different *Plasmodium* species. The most lethal form is caused by *P. falciparum* which has developed resistance to many chemotherapeutic agents and possibly to the current drugs of choice. Reducing the impact of malaria is a key to achieving the sustainable development goals which are geared toward combating the disease. Covalent bitherapy is a rational and logical way of drug design which entails joining a couple of molecules with individual intrinsic action into a unique agent, hence packaging dual activity into one hybrid. This suggests the need to develop new antimalarial drugs that are effective against malaria parasites based on the new mode of action, molecular targets, and chemical structures. In silico studies have shown that sarcosine is able to bind to unique plasmodia proteins (*P. falciparum* ATCase), whereas aniline can be a ligand to target protein (enoyl acyl carrier protein reductase), hence suppressing the progression of the disease. The main objective of this study was to synthesize and determine the efficacy and safety of antiplasmodial hybrid drug comprising the sarcosine and aniline derivative for management of plasmodial infections. The hybrid drug was synthesized by adding thionyl chloride to sarcosine to form acyl chloride which was then added to aniline to form sarcosine-aniline hybrid molecule. The IC50 of sarcosine-aniline hybrid was 44.80 ± 4.70 ng/ml compared with that of aniline derivative which was 22.86 ± 1.26 ng/ml. The IC50 of control drugs was 2.63 ± 0.38 ng/ml and 5.69 ± 0.39 ng/ml for artesunate and chloroquine, respectively. There was a significant difference between IC50 of sarcosine-aniline hybrid and aniline derivative (*p* < 0.05). There was also a significant difference between sarcosine-aniline hybrid and standard drugs used to treat malaria including artesunate and chloroquine (*p* < 0.05). The ED50 of sarcosine-aniline hybrid drug was 6.49 mg/kg compared with that of aniline derivative which was 3.61 mg/kg. The ED50 of control drugs was 3.56 mg/kg, 2.94 mg/kg, and 1.78 mg/kg for artesunate-aniline hybrid, artesunate, and chloroquine, respectively. There was a significant difference (*p* < 0.05) between ED50 of sarcosine-aniline hybrid and both controls such as aniline derivative, artesunate, artesunate-aniline hybrid, and chloroquine. Cytotoxicity results revealed that sarcosine-aniline hybrid was safe to vero cells with a CC50 of 50.18 ± 3.53 *μ*g/ml. Sarcosine-aniline hybrid was significantly less toxic compared with artesunate, chloroquine, and doxorubicin. Sarcosine-aniline hybrid was efficacious and safe to mice. Therefore, covalent bitherapy should be used in drug development for drug resistance mitigation.

## 1. Background

**Figure 1:**
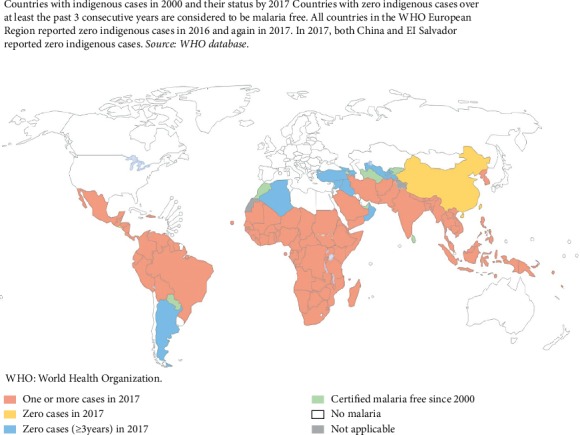
Global distribution of malaria throughout the world (source: World Malaria Report 2018) [1].

Globally, malaria transmission occurs in five WHO regions. It is estimated that 3.2 billion people in 95 countries and territories are at risk of being infected with plasmodium species and developing disease (Figure 1), and 1.2 billion are at high risk, where more than1 in 1000 people have chance of getting malaria in a year. Results from the World Malaria Report of 2015 show that malaria cases were ranging between 149 and 303 million or with an average of 214 million cases of malaria globally. The African region accounted for most global cases of malaria (88%), followed by the Southeast Asian region (10%) and the eastern Mediterranean region (2%). In addition, the number of people who died from malaria was in the range of 236, 000 to 635, 000, representing an average of 438 000 malaria deaths. The high burden was heaviest in the WHO African region (Figure 1), where an estimated 90% of all malaria deaths usually occur (Figure 1), and in children aged under 5 years, who accounted for more than two-thirds of all deaths [1]. This is because children of this age-group are highly susceptible to infection, illness than adults [2]. From the year 2000, malaria incidence rates decreased by 37% globally and by 42% in Africa, whereas malaria mortality rates went down by 66% in the African region and by 60% globally (Figure 1) [1].

Chemotherapy has been the backbone of malaria control strategy [3]. The *Plasmodium* species that are considered responsible for malaria in humans are *P. vivax*, *P. falciparum*, *P. ovale*, *P. malariae*, and *P. knowlesi* [4]. However, *P. falciparum*, the parasite that causes over 90% of all global malaria cases is more frequently becoming resistant to classical antimalarials, necessitating an urgent need for research and synthesis of new antimalarial agents, preferably with novel mode of action [3]. In the past two decades, only a few compounds belonging to a new class of antimalarial drugs, including aminoalcohols such as mefloquine, halofantrine, and lumefantrine; sesquiterpene trioxanes such as artemisinin derivatives; and naphthoquinones such as atovaquone were developed for clinical usage [5]. Currently, artemisinin-based combination therapy (ACT) is considered as the gold standard against *P. falciparum*, in which the regimen uses a double combination therapy geared toward delay of resistance, or preventing it altogether. Many approaches to antimalarial drug discovery deployed include optimization of therapy with available drugs such as combination therapy, developing analogs of the existing drugs, evaluation of potent agents from natural products especially plants, use of compounds originally developed against other diseases, evaluation of drug-resistance reversers (chemosensitizers), and new chemotherapeutic targets [3].

Currently, chemotherapy is the most important strategy to treat and prevent malaria. However, reports from Southeast Asia indicate parasite resistance to artemisinin based combination therapy, which is considered as the gold standard in treatment of malaria according to the WHO [6]. This suggests the need to develop new antimalarial drugs that are effective against malaria parasite based on a new mode of action, molecular targets, and chemical structures. In silico studies have shown that sarcosine (N-methyl glycine) is able to bind to unique plasmodia proteins, hence inhibiting parasite growth. In addition, it was shown that 3-chloro-4-(4-chlorophenoxy) aniline (aniline derivative) can bind to *P. f* ENR (enoyl acyl carrier protein reductase), an enzyme which catalyzes the last step of the elongation cycle in the biosynthesis of fatty acids [7], thereby inhibiting the parasite growth. Nevertheless, no experimental study has been conducted to confirm this aspect of the two to have antiplasmodial activity singly or in a hybrid molecule.

**Figure 2:**
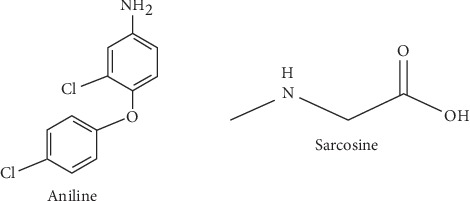
Structure of aniline and sarcosine.

Figure 1 shows that most of the malaria cases occur in Africa followed by Southeast Asia and Latin America. From the same figure, it is also shown that malaria has been eradicated in the USA and Europe. Sarcosine and aniline were suggested to have antiplasmodial activity based on bioinformatics studies. Covalent linking of sarcosine and aniline can carry a dual activity with different modes of action for inhibiting plasmodial growth. Based on the urgent need in drug development, it was necessary to validate this claim in this study. The expected outcome was that sarcosine-aniline hybrid could contain the dual activity of inhibiting the pyrimidine and fatty acid biosynthesis, which might circumvent the parasite resistance to ACTs. Chemical structures of sarcosine and aniline are shown in Figure 2.

## 2. Methods

### 2.1. Study Design

This project was a laboratory-based experimental study carried out at the Kenya Medical Research Institute (KEMRI), Centre for Biotechnology Research and Development (CBRD), Malaria Unit in Collaboration with the Center For Virology Research (CVML) and Center for Traditional Medicine Drugs and Research (CTMDR).

### 2.2. Sources of Chemicals

Sarcosine, aniline, thionyl chloride, dichloromethane, magnesium sulfate, and ammonium chloride, thin layer chromatography (TLC) plates, hypoxanthine, dimethyl sulfoxide, and other supplies were provided by Sigma Aldrich company.

### 2.3. Chemical Synthesis: Procedure for Coupling Aniline to Sarcosine

Thionyl chloride (1.5 mL, 20 mmol) was added to sarcosine (0.09 g, 1.0 mmol), and the resulting suspension was refluxed for 6 hours to give a clear yellow solution. Excess thionyl chloride was removed in vacuo, and the acid chloride was dissolved in dry dichloromethane (CH_2_Cl_2_) (10 mL) and cooled to 0°C. A solution of 3-chloro-4-(4-chlorophenoxy) aniline (4.0 mmol) and triethylamine (0.20 mL, 2.0 mmol) in dry CH_2_Cl_2_ (2.5 mL) was added via a cannula. The resulting mixture was stirred at room temperature for 16 hours during which time a white precipitate was formed. The suspension was washed with half-saturated aqueous ammonium chloride solution (2 × 6 mL) and water (2 × 3 mL), then dried over anhydrous magnesium sulphate (MgSO_4_), and concentrated in vacuo. The formation of a hybrid molecule was monitored by thin layer chromatography (TLC).

The sarcosine-aniline hybrid drug was synthesized by coupling sarcosine to aniline as shown in the following reactions.

**Figure 3:**
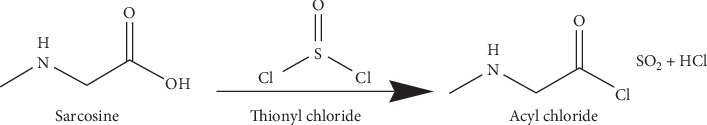
Reaction between sarcosine and thionyl chloride.

Figure 3 shows the reaction between sarcosine and thionyl chloride in dichloromethane solvent to form acyl chloride. This reaction is irreversible because SO_2_ and HCl gasses are lost in the reaction mixture.

**Figure 4:**
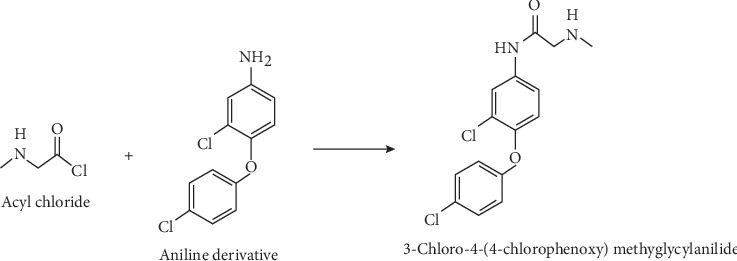
Coupling acyl chloride to aniline.

**Figure 5:**
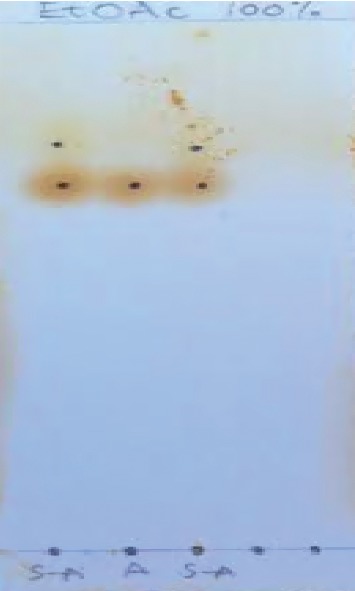
Thin layer chromatography results

Figure 4 illustrates the reaction between acyl chloride and aniline to form sarcosine-aniline hybrid at room temperature. In this reaction, there is a nucleophilic substitution of Cl by NH_2_. The formation of sarcosine-aniline hybrid was spotted on thin layer chromatography and observed as band under UV light (Figure 5).

### 2.4. Thin Layer Chromatography Procedure

TLC was run by spotting the sarcosine-aniline hybrid on the TLC plate in a solvent system of ethyl acetate (2 ml). The plate was dried to observe and take a photo of the spot, in a small container that has a lid mix of about 4 g of silica gel and 1 g of iodine crystals. The TLC plate was placed in the mixture and shook gently to get in contact with the TLC plate for 5 minutes. The plate was taken to fluorescent machine under UV to observe dark spots of aniline and sarcosine-aniline hybrid. After TLC experiment, the location of each spot on the plate was represented by calculating its retention factor (Rf). The retention factor (Rf) was calculated by dividing the distance travelled by the compound by the distance from the baseline to the solvent front [8].

(1) $$\begin{array}{c} \text{Rf}=\frac{\text{distance spot moved}}{\text{distance solvent moved}}. \end{array}$$

Figure 5 shows the results of sarcosine-aniline hybrid formation on the thin layer chromatography plate. EtOAc stands for ethyl acetate, and the solvent system used to run this TLC consisted only of EtOAc (100% concentrated). Aniline was used as a control in the middle of the plate (second column). From the left to right, for the first and third column, the first spots closer to the base line of the columns is 3-chloro-4-(4-chlorophenoxy) aniline, then the spots closer to the top are sarcosine-aniline hybrid. The sarcosine-aniline hybrid is less polar than 3-chloro-4-(4-chlorophenoxy) aniline, so it would elute (released) faster than 3-chloro-4-(4-chlorophenoxy) aniline. The retention factor (Rf) value for the sarcosine aniline hybrid (S-A) was 4.4/5.8 = 0.76, and the Rf of aniline was 4/5.8 = 0.69.

### 2.5. *In Vitro* Bioassays

#### 2.5.1. Drug Solutions and *Plasmodium falciparum* Cultures

Stock solution of the test compound and reference drugs was prepared with sterile water (deionized and autoclaved). The drugs which were insoluble in water were enhanced by first dissolving them in dimethylsulfoxide (solvent concentration <0.02%). The test samples were prepared as a 10 mg·ml^−1^ stock solution. Further dilutions were prepared on the day of biological assays. All the drug solutions were stored at 4°C for later use.

Laboratory-adapted *P. falciparum* strains isolates 3D7 (CQ sensitive) were used in this study. The strains were grown and maintained at the Kenya Medical Research Institute, malaria laboratory. Sterile tissue culture flasks were used which contained complete medium with 1% cell suspension at different initial parasitaemia. The culture medium consisted of RPMI 1640 supplemented with 10% human serum, 25 mM N-2-hydroxyethylpiperazine-N-2-ethanesulfonic acid (HEPES), and 25 mM NaHCO_3_ [9]. Human type O+ erythrocytes (<28 days old), obtained from healthy volunteers after ethical approval and obtaining consent from the participants, served as host cells. The volunteer read and complete the questionnaire of blood donation for *in vitro P. falciparum* cultures which were kept in CBRD/malaria unit and remained private and confidential for each volunteer. The donor was acceptable when he/she is not taking antibiotics or antimalarial drugs. In addition, the blood was tested for HIV and hepatitis antigens at the Center for Virology Research, from which positive samples were immediately incinerated. The accepted blood samples were stored at 4°C in a fridge. The cultures were incubated at 37°C in an atmosphere of 3% CO_2_, 5% O_2_, and 92% N_2_.

#### 2.5.2. *In Vitro* Drug Sensitivity Studies

For measuring the antiplasmodial activity, aliquots (25 *μ*L) of the culture medium were added to all the wells of a 96-well microculture plate. The hybrid drug solution in volumes of 25 *μ*L were added in triplicate, to the first well, and then the serial dilution was carried out to the following wells to make serial dilutions of each sample over an 11-fold concentration range from the highest concentration of 100 *η*g·ml^−1^ to 0.0975 *η*g·ml^−1^. The same procedure was carried out for reference drugs such as artesunate and chloroquine [10].

The quantitative evaluation of antimalarial activity *in vitro* was carried out by using a semiautomated microdilution technique known as tritiated hypoxanthine incorporation assay (THA). Radiolabeled hypoxanthine uptake by the parasite is an indicator of its growth and multiplication. This method helped to determine the antimalarial drug activity against the intraerythrocytic asexual stage of *P. falciparum* grown in the *in vitro* culture [11].

For each microwell solution, 25 *μ*l of [^3^H] hypoxanthine was added, and the reaction was allowed to take place for 48 hours of incubation at 37°C. In this method, the amount of tritiated hypoxanthine, which is incorporated into parasite DNA, was determined by the scintillation counting method. Thus, the determination of labeled tritium hypoxanthine inserted into parasite DNA during replication might be used to define parasite drug susceptibility [11]. After 48 hours of incubation, the parasites were harvested using harvesting machine, and measurement of radioactivity was performed by scintillation counter machine (Wallac1450 Microbeta reader). Mean counts per minute (cpm) are generally in the range of 20,000–60,000, with an acceptable minimum of 10,000 [12].

(2) $$\begin{array}{c} \text{\% reduction in }3\text{H Hypoxanthine uptake}=100\;*\;\frac{\text{gm of no drug sample}-\text{gm of test samples}}{\text{gm of no drug sample}}, \end{array}$$

where gm refers to geometric mean cpm (counts per minute) [12].

Percent reductions in ^3^H Hypoxanthine uptake was used to plot percentage inhibition of growth as a function of drug concentration. IC_50_ activity was determined by linear regression analyses on the linear segments of the dose response curve.

### 2.6. *In Vivo* Bioassays

#### 2.6.1. Experimental Animals and Parasites

Female Swiss albino mice were collected and kept in the animal house of the Center for Biotechnology and Research Development at the Kenya Medical Research Institute (KEMRI), where the animal experiments were carried out. The animals were six weeks old, weighing 20 to 22 g, and they were kept in mice cages and were allowed to access mice pencils (Ungafeed Company Ltd) and water before experimental testing. *Plasmodium berghei* ANKA strains, sensitive to quinolone and artemisinin-based drugs, were obtained from the Unit of Malaria, Center for Biotechnology and Research and Development, Kenya Medical Research Institute. Parasites were maintained and cryopreserved in a freezer (−80°C). The parasite was subsequently maintained in the laboratory by serial blood passage from mouse to mouse on a weekly basis.

#### 2.6.2. Procedure for *In Vivo* Efficacy of Sarcosine-Aniline Hybrid

This consisted of *in vivo* evaluation of hybrid drug against *P. berghei* ANKA, a rodent parasite which is commonly used in antimalarial studies [13]. Female Swiss albino donor mouse was injected intraperitoneally with 0.2 ml of inoculums of 1 × 10^7^ parasitized erythrocytes, and *P. berghei* ANKA strain was obtained from the donor mouse [14]. Parasitaemia was assessed after 5 days under microscope using the Giemsa staining technique. The donor mouse was sedated using carbon dioxide to collect blood via cardiac puncture in a heparinized tube using a syringe and a needle. Carcasses were pooled in a biohazard container and stored at room temperature while waiting to be incinerated. Afterward, the experimental mice were infected with inoculums of 1 × 10^7^ parasitized erythrocytes using an intraperitoneal method. The mice were grouped into five groups consisting of 5 mice per group. The sarcosine-aniline hybrid was dissolved in 10% of Tween 80, and oral drug was administered daily for 4 days. Blood for making thin blood smear was collected from the tip of the mouse tail. The efficacy of the drug was measured by comparison of blood parasitaemia after the four days of therapy (i.e., on day 5 postinfection). The ones still alive at the end of the experiments were killed by using carbon dioxide gas followed by incineration.

The determination of the dose to be administered was calculated individually according to each mouse weight. In this regard, these formulas were applied: HED (mg/kg) = Animal Dose (mg/kg) ∗ [Animal km/Human km], where HED stands for human equivalent dose and km which is a conversion factor. With km (a constant) = Weight/Body surface area. For mice of 0.02 kg, with a body surface area of 0.007, km = 0.02/0.007 = 3 [15].

(3) $$\begin{array}{c} \mathrm{Dosage}\;\left(\text{mg}/\text{kg}\right)\;=\frac{\text{Drug concentration }\left(\text{mg}/\text{ml}\right)*\mathrm{Volume}\;\left(\text{ml}\right)}{\text{Body weight}\left(\text{kg}\right)}. \end{array}$$

From formula (2) [16],

(4) $$\begin{array}{c} \mathrm{Volume}\;\left(\text{ml}\right)\text{ of drug to be given to each mouse}=\frac{\text{Body weight }\left(\text{kg}\right)*\mathrm{Dosage}\;\left(\text{mg}/\text{kg}\right)}{\text{Drug concentration }\left(\text{mg}/\text{ml}\right)\;}. \end{array}$$

Administration of the dose for testing drugs and reference drugs (artesunate and chloroquine) to assess the treatment of the experimental groups had been carried out using an oral method by administering 0.2 ml of infected blood with varying dosages of 10, 5, 2.5, 1.25, and 0.625 mg·kg^−1^. The control groups had been given the normal saline alongside testing using the same procedure. The parasitaemia was checked for every 24 hours from the time of infection for 4 days. Thin blood smears were prepared from each mouse tail venous blood. The smears were prepared by the Giemsa staining technique, and the parasitaemia of individual mouse had been checked using a light microscope [12]. The following formula was used to calculate the percentage parasitaemia [12]:

(5) $$\begin{array}{c} \text{The Percentage of parasitaemia}=\frac{\text{Number of parasitized RBCs}/\text{field}}{\text{Total number of RBCs}/\text{field}}\;*100. \end{array}$$

After 4 days, the percentage (%) chemosuppression of each drug was determined using the following formula [17]:

(6) $$\begin{array}{c} \text{Percentage chemosuppression}=\;100-\left[\left(\frac{A-B}{A}\right)\times 100\right], \end{array}$$

where *A* is the mean parasitaemia in the negative control group and *B* is the parasitaemia in the test group. The dose that cured 50% of infected animals had been determined as effective dose (ED_50_) using a nonlinear regression logistic dose-response model.

### 2.7. Cytotoxicity Assays

#### 2.7.1. Preparation of Drug Solutions and Culture of Vero Cells Used in Cytotoxicity Study

To conduct *in vitro* toxicity, stock solutions of the test compound and reference drugs were prepared with sterile water. The drugs which were insoluble in water had their solubility been enhanced by first dissolving 10 mg in 100 *μ*l of 100% dimethyl-sulfoxide. The test samples were prepared as a 1 mg·ml^−1^ stock solution. Further dilutions were prepared on the day of biological assays. All the drug solutions were stored at 4°C for later use. Vero cells (Vero E-6) were obtained from the Center for Traditional Medicine Drugs and Research (CTMDR), KEMRI. Vero cells are kidney cells extracted from an African green monkey (*Cercopithecus aethiops*). They were stored in a nitrogen tank (−191°C) in the biology department, CTMDR, KEMRI. Cells were maintained in Minimum Essential Eagle's Medium (MEM) containing 10% fetal bovine serum (FBS), PenStrep, and glutamine. Vero cells were cultured using T-75 culture flasks. The flasks were kept at 37°C in 5% CO_2_, and the cells were passaged every 2 to 3 days to keep the cells alive. Trypsinization was carried out to detached cells which overlap each other and counted using a hemocytometer (Neubauer).

#### 2.7.2. Procedure for Cytotoxicity Assay

Using a 96-microwell plate, a cell density of 20,000 vero cells were seeded and incubated for 24 hours at 37°C under 5% CO_2_ to allow cells to attach to the surface/base of the plate. Sarcosine-aniline hybrid drug as the test drug, and controls were added in triplicate to the cultured cells in a concentration range 100 ug/ml, and subsequent dilutions were carried out over 7 folds, from 100, 50, 25, 12.5, 6.25, 3.125, 1.5625, to 0.78125 *μ*g/ml. The plates were incubated for 48 hours at 37°C under 5% CO_2_ to allow the reaction to occur. After 48 hours, 0.5 mg/ml of 3-(4, 5-dimethylthiazol-2-yl)-2,5-diphenyltetra-zolium bromide (MTT) was added for calorimetric measurement of the ability of a drug to kill the vero cell lines [18]. The plates were incubated at 37°C for 3 hours. 100 *μ*L of 100% DMSO was added to each well and mixed to ensure cell lysis and dissolving of the formazan crystals. Doxorubicin was used as a control drug.

Optical density was read using Multi Skan Ex reader machine 48X, from Thermo Fisher Scientific company in a UV–visible spectrophotometry at 562 and 690 nm. The amount of formazan measured was directly proportional to the number of viable cells. The results were recorded as optical density (OD) per well at each drug concentration and analyzed using Microsoft Excel software 2010, from which the percentage of cytotoxicity (PC) was calculated using the following equation: Percentage cytotoxicity = [(*A* − *B*)/*A*] × 100 [19]. where *A* is the mean OD of untreated cells and *B* is the mean OD at each drug concentration. The drug concentration that leads to 50% inhibition of cell growth (CC_50_) was determined by the nonlinear regression logistic dose-response model.

The end points of the mouse experiment in the in vivo assays were set to consider with the development of the clinical signs such as impaired ambulation which prevents animals from reaching for food or water, excessive weight loss and extreme emaciation, lack of physical or mental alertness, difficult labored breathing, and prolonged inability to remain upright. To avoid severe and enduring distress, mice that showed those clinical signs were euthanized by passing carbon dioxide gas into a closed chamber and then incinerating.

### 2.8. *In Vivo* Toxicity Studies

In this step, acute toxicity was performed. Three dosages, such as 2000 mg·kg^−1^, and 300 mg·kg^−1^, and 50 mg/kg were administered orally to mice in three groups, and the fourth group which served as a control received water as a placebo. Each group consisted of 3 mice, and the weight and number of dead mice were monitored and recorded after each 4 days over an interval of 14 days. Clinical or physical signs were also subjected to be recorded during the study period. At the end of the experiment, the mice were killed by using concentrated carbon dioxide gas followed by incineration. The number of dead mice was to be recorded, and a semilog plot of log concentration against percentage (%) response was made. The dose that was to kill 50% of the mice will was to be called LD50 (lethal death) [20], and from this, we can deduce the therapeutic index = LD50/ED50.

## 3. Results

### 3.1. *In Vitro* Activity of Sarcosine-Aniline Hybrid

#### 3.1.1. *In Vitro* Activity of 3-Chloro-4(4-chlorophenoxy) Methyglycylanilde

Table 1 shows the activity of the drug which inhibits the growth of *P. falciparum 3D7* strains, CQ sensitive. The IC_50_ of sarcosine-aniline hybrid was 44.80 ng/ml. Sarcosine did not show *in vitro* activity. Table 1 also describes the comparisons of IC_50_ of sarcosine-aniline hybrid drug against artesunate, aniline, and chloroquine. There was a significant difference between the IC_50_ of sarcosine-aniline hybrid drug against aniline, artesunate, and chloroquine (*p* < 0.05). There was a significant difference between positive controls (artesunate and chloroquine) and aniline. However, there was no significant difference between artesunate and chloroquine (*p* > 0.05).

### 3.2. *In Vivo* Efficacy Studies

**Figure 6:**
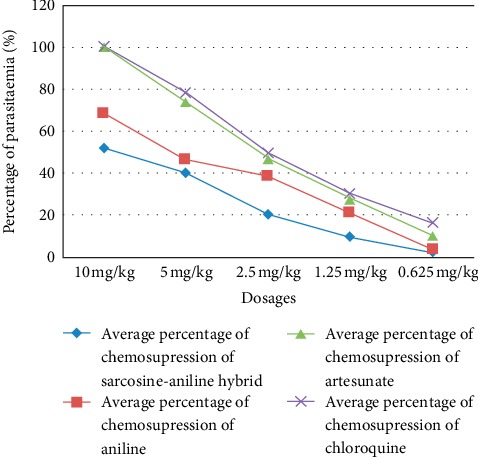
Percentage of parasitaemia chemosupression of sarcosine-aniline hybrid.

Figure 6 shows the percentage of chemosupression of parasitaemia for sarcosine-aniline hybrid, aniline derivative, artesunate, artesunate-aniline hybrid, and chloroquine after 4 days of oral treatment. The percentage of chemosupression for mice treated with 10 mg/kg of sarcosine-aniline hybrid was 52%, whereas those treated with aniline was 68%. No parasites were observed under the microscope for the mice treated with 10 mg/kg of artesunate and that of chloroquine.

Table 2 shows the effective dose of the drug which inhibits the growth of *P. berghei* ANKA sensitive strain to quinoline- and artemisinin-based drug parasites by 50% after four days of oral treatment. The ED_50_ of sarcosine-aniline hybrid was 6.49 mg/kg. Other drugs and compounds such as artesunate, artesunate-aniline hybrid, chloroquine, and sarcosine were used as controls. Sarcosine did not show *in vivo* activity.

Table 2 also describes the comparisons of ED_50_ of sarcosine-aniline hybrid drug against artesunate, artesunate-aniline hybrid, aniline, and chloroquine. There was a significant difference between ED_50_ of sarcosine-aniline hybrid drug against artesunate, artesunate-aniline hybrid, and chloroquine (*p* < 0.05).

Table 3 shows the actual average of parasitaemia at days 7, 9, and 11 after stopping the treatment with 10 mg/kg. The parasitaemia of sarcosine-aniline hybrid was 9.74%, 12.98%, and 15.61% on days 7, 9, and 11, respectively. The recrudescence of CQ and artesunate reappeared on day 9 with 0.03% and 0.2%, respectively.

Table 4 shows the number of mice which survived up to 11 days of experiment, which is 7 days after stopping oral drug administration. For sarcosine-aniline hybrid, 3 mice among 5 survived in the groups which received 10 mg/kg, and 2 mice survived in that group which received 5 mg/kg, whereas only 1 mouse survived in the groups which received 2.5 mg/kg, 1.25 mg/kg, and 0.625 mg/kg. Up to day 11, all mice which received 10 mg/kg of artesunate and chloroquine survived along *in vivo* efficacy testing.

### 3.3. *In Vivo* Toxicity

#### 3.3.1. Acute Toxicity

Table 5 illustrates the number of dead mice during acute toxicity testing for 14 days. The three groups of mice received different dosage (2000 mg/kg, 300 mg/kg, and 50 mg/kg) of sarcosine-aniline hybrid drug, and the control group did not receive any drug but water. For all groups, the initial weights ranged from 20 to 22 g, and they were all females. No mouse died in all groups for 14 days.

#### 3.3.2. Weights Changes and Physical Features during Acute Toxicity Experiment

The weight of each individual mouse in each group and the weight variation in for 14 days of acute toxicity testing were recorded. Three groups of mice received different dosage (2000 mg/kg, 300 mg/kg, and 50 mg/kg) of sarcosine-aniline hybrid drug, and the control group did not receive any drug but water. The initial weights for all groups ranged from 20 to 22 g, and they were all females. The weights were recorded after 4 days of intervals. The mean average of weight for the mice which received 2000 mg/kg was 21.34 g after 14 days. The mean average of weights for the mice which received 300 mg/kg, 50 mg/kg, and mice in the control group were 21.58 g, 22.24 g, and 22.33 g, respectively, after 14 days of acute toxicity testing. There was no significant difference in weight changes between mice which received 200mg/kg, 300mg/kg, and 50mg/kg and between the control groups(p>0.05). There was no critical physical manifestation of pain distress observed during acute toxicity period.

### 3.4. Cytotoxicity Results

Table 6 shows the CC_50,_Standard deviation (SD) of the mean of sarcosine-aniline hybrid drug against vero cells after triplicate experiments. 3-(4,5-dimethylthiazol-2-yl)-2,5-diphenyltetrazolium bromide (MTT) assay was used which is based on the ability of a mitochondrial dehydrogenase enzyme from viable cells to cleave the tetrazolium rings of pale yellow MTT and thereby form dark blue formazan crystals which are largely impermeable to cell membranes, resulting in their accumulation within healthy cell cytoplasms. Chloroquine and artesunate were used as standard drugs which are used to treat malaria, whereas doxorubicin was used as a known cytotoxic drug. During experiment, the starting concentration (highest) for both drugs was 100 *μ*g/ml. Sarcosine-aniline hybrid showed a CC_50_ of 50.18 *μ*g/ml, while the CC_50_ of doxorubicin was 1.96 *μ*g/ml.

If CC_50_ of a drug is less than 2 *μ*g/ml, the drug is considered as cytotoxic, and when CC_50_ is 2–89 *μ*g/ml, the drug is considered as moderately cytotoxic, and when it is above 90 *μ*g/ml, the drug is considered as not cytotoxic (safe) [21].

Regarding CC_50_ of sarcosine-aniline hybrid drug against artesunate, chloroquine, and doxorubicin, there was a significant difference between the CC_50_ of sarcosine-aniline hybrid drug against chloroquine, artesunate, and doxorubicin (*p* < 0.05). The highest mean difference was observed between the CC_50_ of sarcosine-aniline hybrid and doxorubicin (48.21), whereas the lowest mean difference was observed between sarcosine-aniline hybrid and chloroquine (-7.78).

### 3.5. Therapeutic Index of Sarcosine-Aniline Hybrid

The therapeutic index (TI) of drug is equal to its lethal dose concentration divided by its effective dose (LD50/ED50). The LD_50_ of sarcosine-aniline hybrid was estimated to be greater than 5000 mg/kg, and its ED50 was 6.49 mg/kg. Therefore, the therapeutic index of sarcosine-aniline hybrid drug was greater than 770.41.

## 4. Discussion

### 4.1. Sarcosine-Aniline Hybridization

In this study, the two molecules were used to synthesize a hybrid: 3-chloro-4-(4-chlorophenoxy) aniline and sarcosine. The molecular purity of 3-chloro-4-(4-chlorophenoxy) aniline and sarcosine was 97% and 98%, respectively, as confirmed by the manufacturer labels upon their delivery in Kenya by Sigma Aldrich. The sarcosine was completely soluble in water, but the solubility of 3-chloro-4-(4-chlorophenoxy) aniline and the hybrid in water was very low. They were soluble in dimethyl sulfoxide (DMSO) and slightly soluble in Tween 80. It is clear that the low solubility of sarcosine-aniline hybrid affected the drug absorption, distribution, and its metabolism. Confirmed by thin layer chromatography, the hybrid (was successfully synthesized using 3-Chloro-4-(4-chlorophenoxy) aniline and sarcosine pharmacophores.

### 4.2. *In Vivo* Efficacy of Sarcosine-Aniline Hybrid

Results from this study showed that sarcosine-aniline hybrid drug has *in vivo* antimalarial activity. However, sarcosine when used singly was not having inhibition activity against *P. berghei* ANKA sensitive strain (ED_50_ not detectable). This was due to different reasons, such as sarcosine being transformed into glycine by glycine-N-methyl transferase enzyme. The ED_50_ of sarcosine-aniline hybrid drug was 6.49 mg/kg, whereas that of 3-chloro-4-(4-chlorophenoxy) aniline alone was 3.61 mg/kg. There was a significant difference between ED50 of sarcosine-aniline hybrid and both controls such as 3-chloro-4-(4-chlorophenoxy) aniline, artesunate, artesunate-aniline hybrid drug, and chloroquine. On the hand, there was no significant difference between ED_50_ of artesunate-aniline hybrid drug and 3-chloro-4-(4-chlorophenoxy) aniline as well as between 3-chloro-4-(4-chlorophenoxy) aniline and artesunate. In the study carried out on *in vivo* antiplasmodial potentials of the combinations of four Nigerian antimalarial plant extracts, the ED_50_ of CQ using the oral administration pathway was 2.2 mg/kg [22], and this is in agreement with the current study, where the ED_50_ of chloroquine was 1.78 mg/kg.

The *in vivo* activity of sarcosine-aniline hybrid drug was less than that of other control drugs used in this study. This may be due to other conversions of the hybrid drug which may occur along the oral administration pathway such as in the bowels. As a result, this can prevent hybrid molecules from reaching the target sites. The type of the drug administration method for effectiveness of a drug can also be of a great concern. Therefore, if sarcosine-aniline hybrid drug can be administered intravenously or subcutaneously, it might also improve its efficacy and its bioavailability. It is known that artesunate has a shorter half-life, and its combination with other drugs increases its bioavailability. The half-life of both 3-chloro-4-(4-chlorophenoxy) aniline and sarcosine is not yet known to determine their stability while combined or hybridized with other compound or drugs. Artesunate-aniline hybrid drug was active compared with both 3-chloro-4-(4-chlorophenoxy) aniline and artesunate when used singly. This might be because artesunate is a semisynthetic derivative of artemisinin whose water solubility facilitates absorption and provides an advantage over other artemisinins. In addition, artesunate is rapidly hydrolyzed to dihydroartemisinin, which is the most active schizonticidal metabolite [23]. By contrast, 3-chloro-4-(4-chlorophenoxy) aniline being a low water-soluble compound might affect the artesunate solubility in the resultant hybrid drug.

In a study on potent *in vivo* antimalarial activity and representative snapshot pharmacokinetic evaluation of artemisinin-quinoline hybrid, the three synthesized artemisinin-quinoline hybrids differed from each other by one methyl group in the linker and position in the chain; their ED_50_ were 1.1, 1.4, and <0.8 mg/kg, respectively, using the intraperitoneal route and 12, 16, and 13 mg/kg, respectively, using the oral route [24]. The ED_50_ of artesunate was 1.8 mg/kg using the oral route with 80 mg/kg as highest dosage, and it was <1 mg/kg using the intraperitoneal route [24]; these results differ slightly with results of this study because of the dosages and methods used, in current study, and no intraperitoneal method was used rather than the oral method. In another study on oral artesunate dose-response relationship in acute *falciparum* malaria, the effective doses were determined after treating patients from whom the infections were detected with a dose varying from 0 to 250 mg of artesunate together with a curative dose of mefloquine; the resultant ED_50_ was 1.6 mg/kg [25], and this result differs with the current study as the artesunate here was combined with mefloquine, and the study used patients, whereas in this study, *P. berghei* was administered in mice.

The percentage of chemosupression for sarcosine-aniline hybrid was around 55%, whereas that of aniline was 68%, the percentage of chemosupression was 100% for mice treated with 10 mg/kg of artesunate and chloroquine. However, there was a recrudescence after 5 days post-treatment for both mice treated with artesunate and chloroquine, whereas for other drugs, there was a continuous increase in the parasitaemia after stopping the treatment (Table 2). The recrudescence in mice treated with artesunate and chloroquine might be due to some remnant parasites which survived after stopping the treatment. In the study on antimalarial activity of methanolic leaf extract of *Piper betle* L, there was 100% chemosupression for the mice treated with chloroquine with 20 mg/kg using the oral route [26], which agrees with the current study, where the percentage of chemosupression was 100% at day 4 following oral treatment at 10 mg/kg.

The number of mice which survived up to 11 days of experiment during *in vivo* efficacy testing was recorded. Actually, after 7 days when the drug administration was stopped, the number of mice which were still alive was different according to the type of drug and its dosage. This is because at this dosage, there was a 100% of parasite growth inhibition. According to the current study, there was where the low drug dosage showed a high number of survived mice compared with the higher dosage concentration of the same drug.

### 4.3. *In Vitro* Toxicity of Sarcosine-Aniline Hybrid

In-vitro toxicity tests are the alternative method approaches to animal acute toxicity evaluation. Vero cell lines are usually used in prospective studies to determine the cytotoxic effect of different natural and artificial products [27]. Cytotoxicity results from this study showed that the hybrid drug was safe to vero cells. When compared with other standard drugs, sarcosine-aniline hybrid was significantly less toxic compared with artesunate. There was also a significant difference between cytotoxicity of hybrid drug and that of CQ against mammalian cell lines. In the study on effects of chloroquine to inhibit dengue virus type 2 replication in vero cells but not in C6/36 cells, the concentrations equal or greater than 500 *μ*g/ml showed major cytotoxicity but the concentrations equal or lesser than 50 *μ*g/ml did not reveal cytotoxicity effects on vero cells [28]. These results agree with the current study, where the CC_50_ of chloroquine was 57.96 *μ*g/ml.

Therefore, the side effects caused by CQ such as neurotoxicity, leukopenia, retinopathy, and cardiovascular toxicity might be absent, reduced, or increased for sarcosine-aniline hybrid drug, but this requires chronic toxicity to be performed using mice so that blood parameters and histopathology of liver, kidney, heart, and brain dysfunction might be elucidated. Doxorubicin was significantly more toxic than hybrid drug to vero cells. Doxorubicin served as a control in cytotoxicity studies as it was revealed that it is more toxic to cancerous cells and even to normal cells including vero cells which had been used in this study. Doxorubicin's cytotoxicity is based on its capacity to bind to DNA-associated enzymes (topoisomerases), intercalate with DNA base pairs, and target multiple molecular targets to produce a wide range of cytotoxic effects. It also activates the Bcl-2/Bax apoptosis pathway when it interacts with cells' membranes [29].

### 4.4. Acute Toxicity of Sarcosine-Aniline Hybrid

Acute toxicity showed that the hybrid drug was safe to mice. There was no dead mice observed with 2000 mg/kg, so the LD_50_ is expected to be above 5000 mg/kg; thus, this drug is classified in category 5 according to the OECD/OCDE guideline for testing chemical compounds [30]. The weights measured during acute toxicity testing showed that the weight loss was observed in the first week of experiment and increased in the second week of experiment for the group which received 2000 mg/kg and 300 mg/kg. There was a continuous gain in weights for the group which received a single dose of 50 mg/kg and control group. There was no significant difference in weights within and between groups at the end of acute toxicity experiment. No clinical physical signs of discomfort such as impaired ambulation, excessive weight loss, lack of physical or mental alertness, difficulty breathing, prolonged inability to remain upright, and extreme emaciation observed during acute toxicity experiment. The therapeutic index of a drug is the ratio that compares the blood level concentrations at which a given drug is toxic (lethal dose) and the concentration at which the drug is effective (effective dose), which is a vital criterion for drug selection [31].

The therapeutic index of sarcosine-aniline hybrid drug is greater than 770.41, which confirms its safety as the concentration required to cause toxicity (>5000 mg/kg) is far greater than that of required to kill parasites (6.49 mg/kg) [32]. The closer the TI is to 1, the more dangerous is, and the larger the therapeutic index (TI), the safer the drug is [32]. In fact, if the TI is small, the drug might be dosed thoroughly, and the patient receiving the drug should be monitored carefully for any clinical signs of drug toxicity [32]. The study by Xie et al. [31] on new potential antimalarial agents: Therapeutic-index evaluation of pyrroloquinazolinediamine and its prodrugs in a rat model of severe malaria; the therapeutic index of artesunate was 4 [31]. When comparing the results from Xie et al. [31], the therapeutic index of sarcosine-aniline hybrid is 192.6-fold greater than that of artesunate, which implies that the sarcosine-aniline hybrid is safer than artesunate.

## 5. Conclusions

Sarcosine-aniline hybrid has been synthesized using sarcosine and 3-chloro-4-(4-chlorophenoxy) aniline pharmacophores, and the product formation was monitored and confirmed by thin layer chromatography. Sarcosine-aniline hybrid drug is a promising antiplasmodial prodrug as it showed activity for *in vitro* and *in vivo* studies with an IC50 of 44.80 ± 4.70 ng/ml and an ED_50_ of 6.49 mg/kg, which are within acceptable ranges of drugs used to treat severe malaria [1]. This study points out that sarcosine-aniline hybrid drug is safe to vero cells with a CC_50_ of 50.18 ± 3.53 *μ*g/ml compared with doxorubicin which is most toxic with a CC_50_ of 1.96 ± 0.59 *μ*g/ml. The acute toxicity results showed no dead mice up to the dosage of 2000 mg/kg using oral administration for 14 days, and there was no significant loss of weight in mice within and between groups with different dosages of sarcosine-aniline hybrid as well as in the control group. There should be the use of covalent bitherapy in drug development. The use of covalent biotherapy in drug resistance mitigation is recommended.

## Figures and Tables

**Table 1:** IC_50_ of sarcosine-aniline hybrid versus control drugs on *P. falciparum* 3D7 strains.

	Drugs	IC_50_	*p* value			
			1	2	3	4
1	Sarcosine-aniline hybrid drug	44.80 ± 4.70 ng/ml		*p* ≤ 0.001^*∗*^	*p* ≤ 0.001^*∗*^	*p* ≤ 0.001^*∗*^
2	Aniline	22.86 ± 1.26 ng/ml	*p* ≤ 0.001^*∗*^		*p* ≤ 0.001^*∗*^	*p* ≤ 0.001^*∗*^
3	Artesunate	2.63 ± 0.38 ng/ml	*p* ≤ 0.001^*∗*^	*p* ≤ 0.001^*∗*^		0.46
4	Chloroquine	5.69 ± 0.39 ng/ml	*p* ≤ 0.001^*∗*^	*p* ≤ 0.001^*∗*^	0.46	
5	Sarcosine	No activity				

^*∗*^Significant: *p* < 0.05. In this table, 1: sarcosine-aniline hybrid drug, 2: aniline, 3: artesunate, 4: chloroquine, and 5: sarcosine.

**Table 2:** ED_50_ of sarcosine-aniline hybrid versus control drugs on *P. berghei* ANKA.

	Drugs	ED_50_	*p* value					
			1	2	3	4	5	6
1	Sarcosine-aniline hybrid drug	6.49 mg/kg		*p * ^a^	0.1	*p * ^a^	*p * ^a^	*p * ^a^
2	Aniline	3.61 mg/kg	*p * ^a^		*p * ^a^	*p * ^a^	*p * ^a^	*p * ^a^
3	Artesunate-aniline hybrid	3.56 mg/kg	*p * ^a^	0.1		*p * ^a^	*p * ^a^	*p * ^a^
4	Artesunate	2.94 mg/kg	*p * ^a^	0.424	*p * ^a^		*p * ^a^	*p * ^a^
5	Chloroquine	1.78 mg/kg	*p * ^a^	*p * ^a^	*p * ^a^	*p * ^a^		*p * ^a^
6	Sarcosine	No activity	*p * ^a^	*p * ^a^	*p * ^a^	*p * ^a^	*p * ^a^	

*p* ^a^: significant, *p* < 0.05.

**Table 3:** Parasitaemia growth of sarcosine-aniline hybrid after stopping treatment.

Drugs	Day 7	Day 9	Day 11
Sarcosine-aniline hybrid	9.74	12.98	15.61
Aniline	6.97	9.03	11.84
Artesunate	0.00	0.2	0.67
CQ	0.00	0.03	0.42

**Table 4:** Survival rate after 11 days of *in vivo* efficacy testing.

Dosage (mg/kg)	Sarcosine-aniline hybrid	Sarcosine	3-Chloro-4-(4-chlorophenoxy) aniline	Artesunate	Artesunate-aniline hybrid	Chloroquine
10	3	1	4	5	3	5
5	2	2	2	3	2	4
2.5	1	1	3	4	3	4
1.25	1	1	3	3	3	3
0.625	1	0	1	2	1	3

**Table 5:** Acute toxicity in mice following different dosage administrations.

Group	Dosage of hybrid drug administered orally (mg/kg)	Number of mice per group	Number of dead mice after 28 days
Group 1	2000	3	0
Group 2	300	3	0
Group 3	50	3	0
Control group	0	3	0

**Table 6:** The CC_50_ values of the different antimalarial drugs against vero cells.

SN	Drugs	Mean CC_50_ in *μ*g/ml	SD

1	Sarcosine-aniline hybrid	50.18	3.53
2	Chloroquine	57.96	3.85
3	Artesunate	19.69	3.26
4	Doxorubicin	1.96	0.59

*The CC*_*50*_ * values of sarcosine-aniline hybrid versus other antimalarial drugs*			

*SN*	*Cell type to be compared*	*Mean difference*	*p value*
1	Vero sarcosine-aniline hybrid VS vero chloroquine	−7.78	0.006^*∗*^
2	Vero sarcosine-aniline hybrid VS vero artesunate	30.48	*p* ≤ 0.001^*∗*^
3	Vero sarcosine-aniline hybrid VS vero doxorubicin	48.21	*p* ≤ 0.001^*∗*^

## Data Availability

All materials and data used to support the findings of this study are available from the corresponding author upon request.

## Abbreviations

ACT: Artemisinin combination therapy
Aniline: 3-chloro-4-(4-chlorophenoxy) aniline
ATCase: Aspartate carbomyl transferase
CC_50_: Cytotoxicity concentration at 50%
CDC: Center for disease control and prevention
CPM: Counts per minute
CQ: Chloroquine
DMSO: Dimethyl sulfoxide
DNA: Deoxyribonucleic acid
ED50: Effective dose at 50%
ENR: Enoyl acyl carrier protein reductase
GM: Geometric mean
IC_50_: 50% inhibitory concentration
IP: Intraperitoneal
IC_50_: Inhibition concentration at 50%
JKUAT: Jomo Kenyatta University of Agriculture and Technology
KEMRI: Kenya Medical Research Institute
MKU: Mount Kenya University
MTT: 3-(4,5-dimethylthiazol-2-yl)-2,5-diphenyltetrazolium bromide
MEM: Minimum Essential Eagle's Medium
OD: Optical density
OECD: Organization for Economic Co-operation and Development
PAUSTI: Pan African University, Institute of Basic Sciences Technology and Innovation
*P. f* ATPase: *P. falciparum* sarco-endoplasmic reticulum calcium ATPase
*P. f* ENR: Enoyl acyl carrier protein reductase
PMI: President's malaria initiative
RBCs: Red blood cells
SERU: Scientific and Ethics Review Unit
WHO: World Health Organization.
